# Pre- and in-service training of health care workers on immunization data management in LMICs: a scoping review

**DOI:** 10.1186/s12960-019-0437-6

**Published:** 2019-12-02

**Authors:** Edward Nicol, Eunice Turawa, George Bonsu

**Affiliations:** 1 Burden of Disease Research Unit, South African Medical Research Council, Francie van Zjil drive, Parow valley, P.O.Box 19070 Tygerberg, Cape Town, 7500 South Africa; 2 Health Systems and Public Health Division, Faculty of Medicine and Health Sciences, Stellenbosch University, Tygerberg Campus, Francie van Zjil drive, Parow valley, Cape Town, 7500 South Africa; 3 Expanded Programme on Immunization (EPI), Ghana Health Service, Accra, Ghana

**Keywords:** Pre-service training, In-service training, Capacity development, Healthcare providers, Human resources, Workforce, Competences, Immunization, Data management, Curriculum

## Abstract

**Background:**

Healthcare providers (HCPs) are recognized as one of the cornerstones and drivers of health interventions. Roles such as documentation of patient care, data management, analysing, interpreting and appropriate use of data are key to ending vaccine-preventable diseases (VPDs). However, there is a great deal of uncertainty and concerns about HCPs’ skills and competencies regarding immunization data handling and the importance of data use for improving service delivery in low- and middle-income countries (LMICs). Questions about the suitability and relevance of the contents of training curriculum, appropriateness of platforms through which training is delivered and the impact of such training on immunization data handling competencies and service delivery remain a source of concern. This review identified and assessed published studies that report on pre- and in-service training with a focus on HCPs’ competencies and skills to manage immunization data in LMICs.

**Methods:**

An electronic search of six online databases was performed, in addition to websites of the WHO, Global Alliance for Vaccines and Immunization (GAVI), Oxfam International, Save the Children, Community Health Workers Central (CHW Central), UNAIDS and UNICEF. Using appropriate keywords, MeSH terms and selection procedure, 12 articles published between January 1980 and May 2019 on pre- and in-service training of HCPs, interventions geared towards standardized data collection procedures, data documentation and management of immunization data in LMICs, including curriculum reviews, were considered for analysis.

**Results:**

Of the 2705 identified references, only 12 studies met the inclusion criteria. The review provides evidence that shows that combined and multifaceted training interventions could help improve HCPs’ knowledge, skills and competency on immunization data management. It further suggests that offering the right training to HCPs and sustaining standard immunization data management is hampered in LMICs by limited or/lack of training resources.

**Conclusion:**

Pre-service training is fundamental in the skills’ acquisition of HCPs; however, they require additional in-service training and supportive supervision to function effectively in managing immunization data tasks. Continuous capacity development in immunization data-management competencies such as data collection, analysis, interpretation, synthesis and data use should be strengthened at all levels of the health system. Furthermore, there is a need for periodic review of the immunization-training curriculum in health training institutions, capacity development and retraining tutors on the current trends in immunization data management.

## Introduction

Human resources for health, also known as the health workforce, is one of the building blocks proposed by the World Health Organization (WHO) Health Systems Framework and is central to achieving universal health coverage (UHC) and enhancing the achievement of the Sustainable Development Goals (SDGs) [1, 2]. However, concerns about the quality and readiness of the health workforce in low- and middle-income countries (LMICs) in strengthening the health system have been raised. Healthcare providers (HCPs) are the drivers of health interventions; yet, some lack the necessary training and skills to adequately perform the tasks of responding to the health needs and expectations of the population, particularly in LMICs [3, 4]. Therefore, appropriate and adequate training is fundamental for developing competent HCPs who are equipped with the knowledge and skills necessary to deliver quality health services.

High-quality vaccine coverage and vaccine-preventable disease (VPD) surveillance data are needed to monitor the performance and impact of the Expanded Programme on Immunization (EPI), which aims to reduce infant and child mortality and morbidity. However, inconsistent and inaccurate recording and reporting of these data limit the ability to accurately monitor EPI coverage and remain a source of concern particularly in LMICs. Due to poor and sub-standard measurement and errors in vaccination records, reliability and validity of reported data on immunization coverage are highly questionable. Additionally, incomplete and incorrect data entry and poor documentation of coverage, as well as double entries by HCPs, have decreased the reporting quality [5–7]. Capacity building of HCPs on data collection, management and analysis is key to ending VPDs.

Attempts to bridge the gaps associated with poor data quality, which are often attributed to insufficient technology rather than the people who drive the system, have led to the implementation of complex health information systems (HIS), including web-based solutions. Nevertheless, these still require human capacity and capability. Persistent challenges identified with these systems include inadequate human resources, insufficient capacity of HIS staff at all levels of the health system, high staff-attrition rates, inadequate training, unstandardized job descriptions, limited HIS development planning and lack of established HIS career path and accredited training programmes [8].

Competency can be defined as a combination of knowledge, skills and abilities needed to perform a specific task in a given context [9], which can be gained through experience, pre- and in-service training and the assistance of mentors and preceptors [5]. Worldwide, the training curriculum for HCPs is geared towards building professional competencies, through either pre- or in-service or on-the-job training or through healthcare service quality improvement programmes [10]. Pre-service training are recommended pre-qualification curriculum-based training, such as the EPI Prototype Curriculum for nursing/midwifery schools in the WHO African Region [11] and the Mid-Level Management Course for EPI managers [12] prescribed by regulatory bodies for preparing professionals/workers during their initial education and for certifying professionals for practice [13]. While in-service training is a regular process to refresh and update skills, competence and knowledge in key areas relevant to a focused healthcare field and are particularly essential to ending VPDs.

There is a great deal of uncertainty and concerns about HCPs’ skills and competencies regarding immunization data handling and the importance of data use for improving service delivery. It is unclear whether HCPs’ training curriculum and modalities are appropriate and optimal for improved skills and competencies in data collection procedures and management [14]. Questions about the suitability and relevance of the curriculum content, appropriateness of the platform through which the training is delivered and the impact of such training on immunization data handling competencies, optimal knowledge and skills in immunization data quality, records, supervision and management and service delivery, particularly in developing countries, are of concern [15].

This scoping review seeks to identify and assess published studies that report on pre- and in-service training with a focus on healthcare providers’ competencies and skills to manage immunization data in LMICs. For the purpose of this study, we assessed available interventions (on improving immunization data documentation and reporting), pre- and in-service training and compared reports on which modality best informs good-quality immunization data collection and management in LMICs.

## Methods

### Study design

We conducted an exploratory research and systematically mapped available literature on specific topics relating to HCPs’ competencies in immunization data management. Key concepts of pre- and in-service training were identified, and sources of evidence and gaps in the research were also highlighted [16]. This approach was necessary due to the diverse knowledge base of the topic of interest, as well as the broad research objectives. The review was based on the systematic scoping review methodological framework designed by Arksey and O’Malley [17]. Key steps followed included identifying the research objectives, identifying relevant studies, selecting appropriate articles, extracting the data and summarizing and reporting the findings.

### Study population

All categories of health personnel qualified as healthcare providers (HCPs) involved with immunization data management, such as doctors, pharmacists, nurses, midwives as well as community HCPs were included.

### Intervention

In addition, studies on interventions that include any training or capacity-building activities related to standardized data collection procedures and management of immunization data, including curriculum reviews, were considered. As the purpose of this study was to assess and compare pre- and in-service training of HCPs on standardized data collection procedures in immunization services, we assessed interventions that were measured and evaluated. Hence, the study design and outcomes of included studies were left intentionally broad, and a meta-analysis was not appropriate at this stage.

### Inclusion and exclusion criteria

Published articles on pre- and in-service trainings of HCPs, healthcare professional curriculum, on-going training and interventions geared towards standardized data collection, correctness, consistency, data reporting and recording and management of immunization data were included. We also included all studies assessing the effect of pre- and in-service training of HCPs in data management improvement, including data collection, entry, completeness, accuracy and timeliness, as well as training on standardized data collection and maintenance in immunization services, interventions on capacity building, improving skills and competencies in data collection and management in immunization services. All studies evaluating educational curriculum, pre- and in-service trainings of HCPs; capacity development on immunization data competencies in data recording and reporting, data analysis, interpretation, synthesis and presentation of immunization data; and interventions and on-going capacity development on standardized data collection and management in immunization services in LMICs were included.

In addition, studies on HCPs’ responsive training programmes on immunization data and district-level data improvement programmes conducted in LMICs according to the World Bank Group 2012 classification [18] were included. Studies with no primary data collection, on the topic of interest but not original, full-text, research studies, e.g. commentaries, letters, opinion pieces and study protocols or abstracts only, and those on data quality audit and studies not conducted in LMICs were excluded.

### Search strategy and data sources

Comprehensive search terms and strategy based on the ‘Population–Concept–Context (PCC)’ framework for scoping reviews [19] were developed with the help of an information specialist and librarian to identify relevant studies reporting on the pre- and in-service training of HCPs on immunization data management in LMICs (Table 1).

**Table 1:** PCC framework for selecting inclusion criteria

Population	Context	Concept
Healthcare workers, healthcare providers, healthcare professionals, healthcare staff, healthcare practitioners, health workers, providers, healthcare, health professionals, health staff, health practitioners, staff development	Low- and middle- income countries, low-income countries, middle-income countries, poor countries, LMICs, developing countries, under-developed countries, Asia, Africa, Latin America	Training, pre-service training, in-service training, healthcare professional development, health staff development, continuing medical education, health worker competence, health worker performance, health personnel education, healthcare personnel training, data management competencies, data collection, data correctness, data completeness, data quality, data accuracy, data management, data curation, immunization, immunization schedule, immunization, expanded programme on immunization, immunization programme, vaccination

The search terms included keywords and Medical Subject Headings (MeSH) together with corresponding synonyms and associated terms, which are summarized in Table 2. This search strategy was adapted for each of the following electronic databases: PubMed, Web of Science, Cochrane Library, Cumulative Index of Nursing and Allied Health Literature (CINAHL) and BMC Medical Education to retrieve relevant articles published from 1 January 1980 to 31 May 2019. In addition, we searched for relevant grey literature in Google Scholar (first 100 citations). Websites of the WHO, the Global Alliance for Vaccines and Immunization (GAVI), Oxfam International, Save the Children, Community Health Workers Central (CHW Central), UNAIDS and UNICEF were also searched for relevant studies. Websites of organizations working on interventions to improve health worker performance in LMICs and reference lists of identified articles were also searched to identify relevant studies for inclusion.

**Table 2:** Search terms and strategy

Databases	Search terms
PubMed	Query
Search #6	Search (#4 AND #5)
Search #5	Search (Low income country OR low-income countries OR middle - income countries OR LMICs OR “developing countries” OR “low income countries” OR Asia OR Africa OR “Latin America”)
Search #4	Search (#1 AND #2 AND #3)
Search #3	Search (immunization[mh] OR immunisation[tiab] OR immunization[tiab] OR vaccination[tiab] OR vaccination)
Search #2	Search ((training OR pre-service training OR pre-service training[tiab] OR in-service training[tiab] OR inservice training[mh] OR healthcare professional development OR health staff development OR health staff development[tiab] OR continuing medical education OR health worker competence OR health worker performance OR Health personnel education OR Healthcare personnel training OR data management competencies)
Search #1	Search (healthcare workers OR healthcare providers OR healthcare professionals OR healthcare staff OR healthcare practitioners OR health workers OR health providers OR health professionals OR health staff OR health practitioners OR health-care workers OR health-care providers OR health-care professionals OR health-care staff OR health-care practitioners OR Staff development OR Staff development[tiab])

### Study selection

Articles were considered relevant if they were about healthcare providers’ capacity development on data collection and quality in immunization programmes in LMICs. All citations were imported into Thomson Reuter EndNote X7 software, and duplicate citations were removed manually. A two-stage screening process for eligibility was conducted, and articles that met the inclusion criteria were selected. In the first stage of screening, two authors (Eunice Turawa (ET) and Edward Nicol (EN)) independently reviewed titles and abstracts of the citations to identify relevant articles. These results were then compared, and discrepancies resolved. As part of the second-stage screening for review, the full text of all potential eligible studies were retrieved, and two authors (ET and EN) independently reviewed the articles for eligibility for inclusion in the review; discrepancies were resolved through discussion, and when consensus was not reached, the third author (George Bonsu (GB)) intervened. Studies that did not meet the eligibility criteria were excluded at this stage. Articles that could not be obtained through online databases were also excluded from the final analysis. A data extraction form was developed according to pre-specified eligibility criteria. To ensure the reliability and consistency of the extraction form, two authors (EN) and (ET) independently piloted the extraction form using the first 20 articles. Results were compared, and the extraction form amended accordingly. At every stage of study selection and data extraction, discrepancies were resolved through discussion and contribution from the third author (GB).

### Charting and collating the data

Using a pre-designed data-charting form, key and relevant information was identified and systematically extracted from each article that met the inclusion criteria. Following the transfer of the data into an excel spreadsheet, two authors (EN and ET) reviewed the extracted information and selected key focus areas for this review and categorized the information according to the training modules, method of assessment and outcome assessed. The two review authors grouped the outcomes into thematic heading, using the following categories: type of training, authors and titles, study setting, population, method of training and outcome of training (comments/relevant findings). Other useful available information in each article was extracted for clear and deeper understanding of the study.

Data were summarized according to key components and definitions used for categorizing data. For each study, we categorized training or interventions evaluated and the outcomes into four levels (reaction, learning, transfer and results) based on the Kirkpatrick model [20].

## Results

**Fig. 1:**
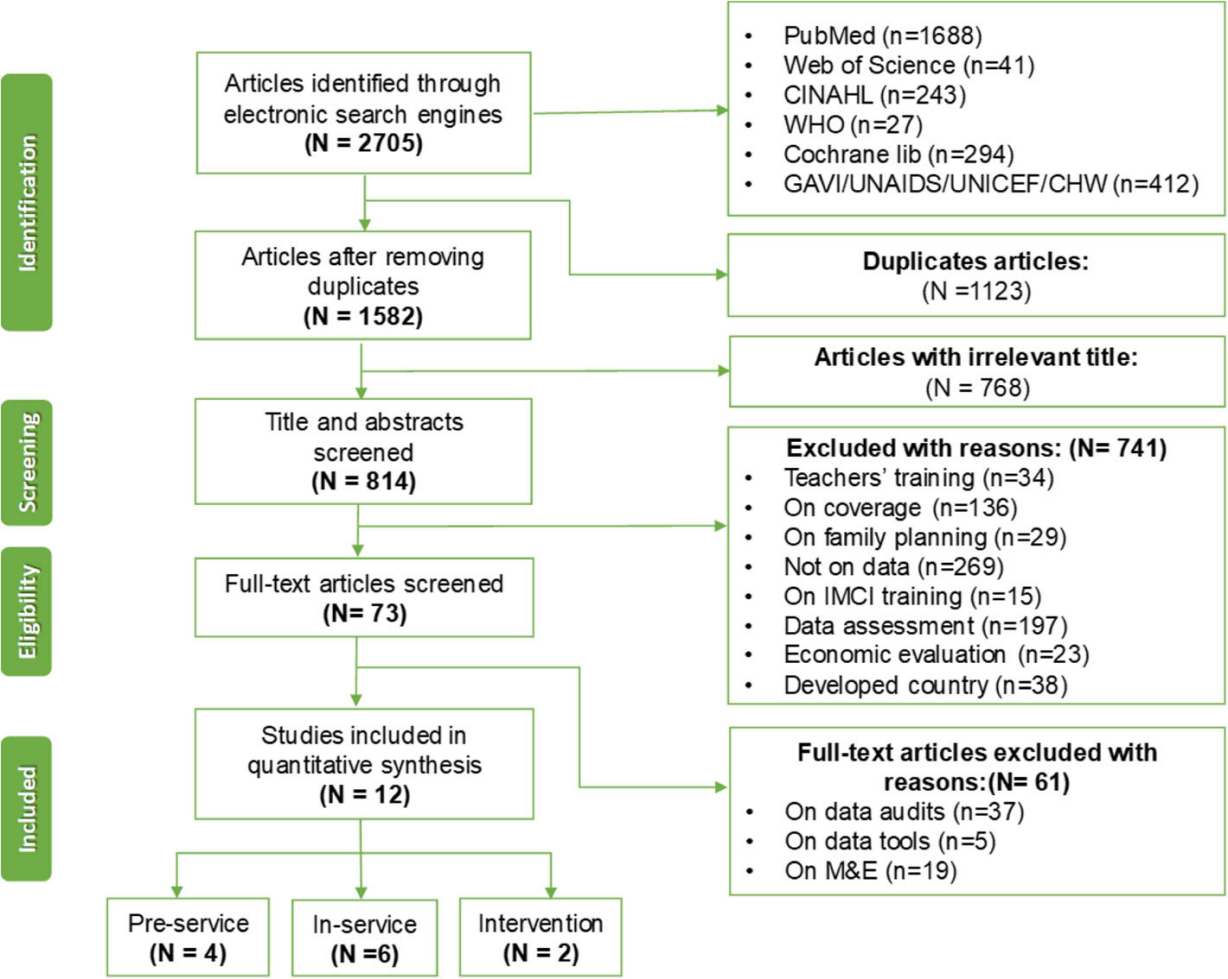
Study inclusion flow diagram

As shown in Fig. 1, the systematic database searches retrieved 2705 articles, of which 1123 duplicate articles screened by titles were excluded using the EndNote referencing system and 1582 articles were independently screened for relevance, leaving a total of 814 articles retained for further assessment. The titles and abstracts of the retained articles were independently screened by two review authors (EN and ET) to identify eligible articles. The full text of 73 articles were reviewed using inclusion and exclusion criteria. Of these, 58 were excluded with reasons (Fig. 1), leaving a total of 12 articles meeting the inclusion criteria. The characteristics of the selected articles are summarized in Table 3.

**Table 3:** Selected pre-service and in-service training in LMICs

Training module	Author and year	Study setting	Population	Intervention(s)/instrument/tools used	Comments/(relevant findings)
Pre-service training	Mutabaruka et al. (2010) [21]	26 training institutions in five selected countries: Ethiopia, Ghana, Lesotho, Senegal and Zambia.	• 642 Mid-Level Management (MLM) Inter-country and Regional offices	• Review of curricula at regional and country levels; Review of WHO/AFRO MLM modules, related reference documents and handouts; country visits for qualitative data collection and analysis • Interviews with past MLM course participants, facilitators, supervisors, Ministry of Health officials, country-based partners and other stakeholders • Focus group discussions with past course participants and facilitators; questionnaires sent to past MLM course participants and facilitator	• Immunization data management not directly referenced • The MLM training increased the performance of trained staff and therefore contributed to the improvement of EPI data management.
Pre-service training	Hossain et al. (2017) [22]	Nursing education institutions: 14 pre-service education institutions (seven public, three faith-based, four private), 23 field placement sites and 29 health facilities	• 14 nursing tutors at pre-service education institutions, 28 nursing graduates in health facilities, 21 health facility managers and 16 sub-county EPI supervisors	• Updated Expanded Programme on Immunization Manuals in the pre-service education institutions	• Limited EPI demonstrations in the classrooms and skills laboratories • Inadequate practical training in documentation and calculation of estimates. Students lacked required knowledge and skills to prepare summary reports and calculate dropout rates. • Graduates of public institutions (KMTCs) appeared to be more competent than those from the private or faith-based universities • Only nine practicum sites (39%), showed students how to calculate coverage rates and plot them on the monitoring chart. • More than half (52%) of the managers thought that nurses were capable of performing EPI activities without in-service training.
In-service training (Capacity building of tutors of pre-service and in-service training institutions)	Mutabaruka et al. (2005) [23]	African Region (12 of the 13 countries covered by the United Nations Foundation (UNF), USAID and the Network for Education and Support in Immunization (NESI)-funded projects)	• Tutors and lecturers at health worker training institutions in Cameroon, Madagascar, Malawi, Mali, Niger, Nigeria, Democratic Republic of Congo, Senegal, Tanzania, Uganda, Zambia and Zimbabwe	• Semi-structured questionnaires (based on a tool developed by WHO/AFRO) were used for interviews with selected key personnel at regional, district and hospital levels, supervisors and health workers, trainers and trainees at pre- and in-service training institutions • Refresher and orientation workshop	• Generally, EPI content was either not outlined in the curricula or was incomplete or outdated • Reference materials and demonstration equipment were also lacking • Time allocated to EPI theory was inadequate and not standardized • Inadequate practical and supervision. Lecturers and tutors lacked modern EPI training.
In-service training (Pre-service and in-service training tutors of health worker)	Umar et al. (2011) [24]	Sokoto and Kebbi States, Nigeria	• Tutors in all the five pre-service health training institutions in Sokoto and Kebbi states of Nigeria. (Training nurses, midwives, community health officers (CHO), community health extension workers (CHEW), senior community health worker (SCHEW) and junior community health worker).	• Questionnaire on bio-data of health workers’ tutors, their highest educational qualification, years of service, in-service training received on EPI, • The five components of Reaching Every Ward (REW) namely planning and management of resources, establishing or reactivating fixed post and outreach services, supportive supervision and community linkage and monitoring for action.	• Although in-service training demonstrated significant statistical association on overall knowledge of respondents’ (df = 1; *F* = 8.62; *P* < 0.0001), less than half of the respondents had received an in-service training on EPI after graduation. • There is a need for retraining of tutors on the current trends in immunization and management.
Pre-service curriculum	Mumbo et al. (2015) [25]	Kenya	• 14 institutions from 18 institutions identified for initial collaboration with the project towards strengthening health workforce training	• The assessment collected data from 533 respondents. • The assessment questions relating to quality of curriculum sought responses on the following: availability of curriculum guidelines, curriculum responsiveness to institutional mission and regularity of curriculum review as well as involvement of stakeholders in the review process	• The findings revealed major gaps in quality and adequacy of curricula in the training institutions. • A national standard framework to guide curricula review process is lacking • Curricula did not adequately prepare students for clinical placement, as most failed to directly respond to national health needs.
In-service training	Weeks et al. (2000) [26]	Kyrgyzstan, Central Asia	• Medical workers (41 health facility staff). • Supervisors of health information records. • Duration of training: two days	• Simplify DPT records and reports, collect only relevant reports and decentralized decision making. • Modify the existing data collection forms and records, developed graphs that could be constructed manually. • Analytic supervision checklist developed for the 3 levels of supervision.	• HCW training and data management • 95% improved data collection and data reporting and quality.
In-service training (Evaluation of dashboard)	Poy et al. (2017) [27]	6 African countries: Angola, Chad, DR Congo, Ethiopia, Nigeria, South Sudan	• Immunization programme managers	• Creation and implementation of multidimensional monitoring tool (i.e. dashboard) designed • to provide information on immunization system performance	• The capacity building workshop and job aids development facilitated the dashboard reporting process clarifying both indicator definitions and reporting timelines • Data availability and quality improved between the first quarter of 2014 and fourth quarter of 2015, especially for the process indicators.
In-service training	Ward et al. (2017) [28]	Kampala, Uganda	• Data improvement team (DIT) between November 2014 and September 2016, all 112 districts and five divisions of Kampala (total 116 DIT operational districts) in Uganda sent staff to DIT regional training and deployed district-level DITs. • Seventeen regional trainings, covering 2–14 districts per training, • Attended by 451 district and health sub-district staff and 35 MakSPH students (some attended multiple trainings)	• Pre-intervention 5-day orientation to the strategy and Uganda’s immunization information systems was provided to national staff. • 3-day regional training aimed to build selected DIT members’ knowledge and skills in data management and quality. • Training was evaluated through a self-administered survey focused on quality of the training experience; a pre-test and post-test measured participants’ acquisition of knowledge and level of preparedness to implement DIT activities. • Organizational assessments contained a mix of closed and open questions covering dimensions of, and factors affecting vaccination data quality. • DITs also used a data quality improvement (DQI) questionnaire to review practices for data management, collection, accuracy, analysis and use	• After training, 83% (355/429) of district staff demonstrated improved knowledge on post-test compared with pre-test scores, and more participants felt ’fully prepared’ to conduct DIT activities (14% pre-test, 82% post-test).
In-service training	Pappaioanou et al. (2003) [29]	Bolivia, Cameroon, Mexico and the Philippines	• Interdisciplinary, in-service training programmes, tailored to the needs of mid-level policymakers, programme managers, technical experts and information specialists,	• National trainers, with support from CDC technical advisors, conducted local training and information needs assessments. Based on needs assessments and post-training job responsibilities of participants, learning objectives were developed for specific target audiences (e.g. decision makers and technical advisors), and training curricula and materials, using information from the country’s HIS. • Workshops include training exercises aimed at teaching priority setting, health problem analysis, programme planning, monitoring and evaluation. Staff were trained, and technical assistance was provided in the use of the HIS and software as part of the interdisciplinary training programmes. • Post-intervention: Existing sources of information and the effectiveness of routine data collection procedures were evaluated for their potential to provide the needed information.	• Results showed that the DDM strategy improved evidence-based public health. • Subsequently, DDM concepts and practices have been institutionalized in participating countries and at CDC.
In-service intervention	Vasan et al. (2017) [30]	LMICs	• Frontline non-physician HCWs in LMICs	• Of the 40 papers, 13 papers used cross-sectional survey study designs based on pre-/post-intervention plausibility design. Eight involved qualitative research using a variety of methods (realist evaluation, focus group discussion, key informant interviews). Seven described randomized controlled trials (RCTs) or studies nested within RCTs, and another seven were reviews, three of which were systematic reviews. Other study designs included were four programme evaluations (using routinely collected data not under research conditions) and one time-use study. • The reviewed studies were categorized under ‘Supervision or Supportive Supervision (23),’ ‘Tools, and Aids, (10)’ ‘Mentoring or Clinical Mentoring’ (3) and three under ‘Quality Improvement (3),’ and ‘Coaching and Peer Review Strategies’ (1)	• The variety of study designs and quality/ performance indicators precluded a formal quantitative data synthesis. • The most extensive literature was on supervision, but there was little clarity on what defines the most effective approach to the supervision activities themselves, let alone the design and implementation of supervision programmes.
In-service intervention (Health Care Provider Performance Review)	Rowe et.al. (2018) [31]	LMICs	• The Health Care Provider Performance Review (HCPPR)	• Systematic review of strategies to improve health-care provider performance in LMICs	• Training combined with supervision or group problem solving strategies tended to be more effective in improving HCW’s performance. • Monitoring HCWs’ performance by implementing evidence-based strategy alongside including local context knowledge likely to be more effective. • Identify and address gaps modifying or remove strategies or adding new strategies. • The effects of any strategy should be monitored so that managers can know how well it is working. • Strategies that include community support plus training for HCWs prove to be more effective in improving healthcare providers. • Use more standardized methods.
Pre-service and in-service training	Tsega et al. (2014) [32]	Health training institutions in Malawi	• Health care professionals (National EPI managers, immunization officers, district health officers, immunization coordinators and supervisors)	• Tailored questionnaires were used to elicit information from central, zonal and district healthcare facilities and training institutions. • At the central, zonal and district levels, interviewees included heads of health offices; at the healthcare facility level, interviewees included immunization coordinators and supervisors	• Graduates not well equipped to provide quality service • Insufficient time allocated for immunization training (50% of school principals) • Half of training institutions had copies of the national immunization guidelines, but had outdated curricula • 50% of the principals said students will benefit from in-service training to be able to perform immunization activities, and reported suboptimal relationship between service providers and training institutions • Insufficient training materials for in-service mid-level management (MLM) training, Electronic copies are only accessible to a few people.

Key concepts of all studies that met the inclusion criteria were systematically summarized to provide a comprehensive but concise descriptive map of the nature and breadth of research on pre- and in-service training of HCPs’ competence on immunization data handling and to identify obvious research gaps. The research objectives were used to identify and evaluate the evidence gaps. All 12 included studies were from LMICs; four studies were from sub-Saharan Africa, one from Asia and five were multi-country studies including Angola, Bolivia, Chad, the Democratic Republic of Congo, Ethiopia, Cameroon, Ghana, Lesotho, Madagascar, Malawi, Mali, Mexico, Niger, Nigeria, Senegal, South Sudan, Tanzania, Uganda, Zambia, Zimbabwe and the Philippines (Table 3). The participants were mainly mid/high-level healthcare officers, including mid-level policymakers, programme managers, technical experts, information specialists, health professional tutors, nurses, nursing students and Expanded Programme on Immunization (EPI) supervisors at health facility, sub-county, inter-country and regional levels. The study focus was on components and contents of healthcare education curriculum, HCPs’ curriculum and assessment of HCPs’ competence on immunization data handling.

### Pre-service training

One third (33%) of the included studies [21, 22, 24, 32] examined pre-service training of HCPs through evaluation of the curriculum (focusing on the new EPI), the knowledge and competency of the tutors and clinical instructors. The studies show significant gaps in knowledge and skills of the tutors. The majority of the tutors and clinical instructors lack modern EPI training methods, and their competency and skills are insufficient to enable adequate translation of knowledge [21, 22, 24]. Furthermore, most of the training institutes (public, faith-based or private) did not have the EPI manual, and where this is available, it is either incomplete or out-dated; hence, the institutions are not adequately equipped to train personnel. Insufficient teaching materials [32], reference books and access to EPI publications were common in most of the training institutions.

Sub-standard or lack of knowledge on supervision and monitoring is a major problem among the tutors. While basic knowledge on the principles of immunization is above average, student clinical practice was poorly supervised due to clinical supervisors’ lack of skill and knowledge. One of the studies showed that only three of the seven medical training colleges in Kenya have the updated WHO EPI prototype curriculum [22], and that only 39% and 64% of the nurse tutors respectively demonstrated to the students how to calculate coverage rates and plot them on the monitoring chart, and evaluated them according to the learning objectives at the end of the class. The studies found that nursing students did not gain sufficient knowledge and skills in the area of EPI documentation (estimating EPI targets, calculating coverage and dropout rates and plotting coverage monitoring charts), since the time allocated for immunization training was insufficient [22, 32], and that newly graduated nurses arrived at their jobs with inadequate knowledge in the areas of EPI documentation and needed on-the-job training and mentorship for at least 1 or 2 weeks after their arrival [22, 32]. The study also found that the level of competency among graduate nurses depended on the educational institutions attended. Only two studies reported on stakeholder involvement [22, 32], and in many of the institutions, the EPI stakeholders were not involved in developing the curriculum.

### In-service training

Nine of the 12 identified studies were on in-service training [23, 24, 26–32]. The majority of which focused on filling the current gaps in practice, need assessments and identifying why, how and what the problems are with data entry, data collection and use of the health information system and software use in management of immunization data [27, 29]. Others focused on pre- and post-training assessment [24, 28, 32], or evaluation of monitoring and evaluation staff (interdisciplinary, in-service training programmes, tailored to the needs of mid-level policymakers, programme managers, technical experts and information specialists). Implementation of post-training changes and improvements were attainable; however, comprehensive EPI training according to the curriculum was comparatively poor in the in-service compared to pre-service training.

#### Training curriculum and tools

One of the major problems in HCP training is the lack of appropriate equipment and teaching aids (e.g. appropriate forms, computers, technologies) and updated reference manuals and curriculum (EPI prototype curriculum and manual). Evidence shows that the majority (75%) of the pre-service training institutions lacked the necessary equipment and tools needed for practical demonstrations and knowledge of current EPI theory and practice [22].

This review found that the curriculum, especially in pre-service institutions, is not responsive to current needs and is often outdated and usually only updated every 5 years [21–23, 30, 32]. This situation is worse in private and faith-based health organizations. Some training institutions do not have EPI in their curriculum; others had incomplete or outdated information leading to outdated policies [23, 26]. Although, most training tutors and lecturers demonstrated sufficient knowledge and skills for teaching immunization data content, they need to be exposed to regular and continuing medical education to ensure up-to-date knowledge in immunization data management. The time allocated for classroom teaching appears to be adequate but the time allocated for practical sessions is insufficient to allow transfer of knowledge and skills in pre-service health provider’s institutions. Another concern from the reviews is the lack of institutionalized collaboration between training institutions and EPI programme managers, which seems to widen the gap in knowledge and adversely affects the quality of theoretical and practical training and HCPs’ skills and competencies. Above all, the linkage between pre- and in-service training is too weak, and the challenges between these two training strategies and performance are difficult to address.

Although Hussein et al. [22] reported hands-on experience on monitoring coverage (estimation of target population, calculation of vaccination coverage, dropout rates and capturing of immunization coverage data into the monitoring chart), the EPI manual was not available to the students because the trained mid-level managers (MLM) failed to circulate the prototype curriculum to the training institutions [32]. Reports also demonstrated that most of the health facility managers did not maintain proper immunization records because they lacked the necessary knowledge and skills required to prepare and complete summary reports and to calculate immunization indicators and dropout rates. Due to the competence and skills gaps in data handling, relevant data are partially documented/not recorded across all tiers of the healthcare setting. Largely, nearly three quarters (72%) of the new graduates did not know how to estimate the immunization (EPI) target population, and about three quarters (70%) did not know how to calculate vaccine-coverage and dropout rates. Additionally, almost all the new HCPs did not know how to plot coverage rates on the coverage monitoring chart. It cannot be overemphasized that essential to good-quality immunization data is the practice of routine data monitoring at all levels of healthcare, as well as the use of simple monitoring tools (charts, mobile devices) that can be incorporated into the hospital health information system resulting in more-reliable data [22, 30].

Health information systems (HISs) are recognized as a useful tool in improving data management and access to essential data from multiple sources, but lack of the required skills to operate HIS and convey data information to the target audience has been a major bottleneck among immunization HCPs [29]. Ward et al. [28] identified gaps in awareness and processes and evaluated the accuracy of immunization data. Inaccuracy in data was observed mainly at the health facility levels and was traced to deficiencies in HCPs’ knowledge and skills in data quality, scarcity of standard recording and reporting tools and inadequate implementation of recommended practices for data collection, analysis and use. They, however, recommended an in-service training module with the help of data improvement teams (DITs) as an intervention to improve the quality of immunization data handling/management. This approach facilitated and strengthened data management analysis and use of data in district health offices and health facilities. Evidence shows that 83% (355/429) of district staff who participated in the in-service training demonstrated improved knowledge on post-test compared with pre-test scores, and more participants felt ‘fully prepared’ to conduct DIT activities (14% pre-test, 82% post-test).

Although routine follow-up and accountability are still a challenge, monitoring and evaluation of HCPs’ activities, supportive supervision and development of specific, measurable, achievable, realistic and time-bound targets are essential for improving health providers’ knowledge and competencies.

### Supportive supervision and monitoring

Supervision can also lead to more efficient and effective data handling by HCPs. Effective supervision is difficult to achieve because there is no clarity on the most effective approach to supervision activities and the design and implementation of supervision. There is also no comparative intervention strategy that establishes a clear standard for performance, quality assessment and improvement in immunization data management [22, 30]. Even though visits are carried out in health facilities, the required supportive supervision is done infrequently and therefore not effective. Many newly qualified HCPs clamour for in-service training and mentorship because they lack adequate knowledge on immunization data-handling skills (specifically calculation of dropout rates and correctly plotting immunization charts). Although the majority of the newly graduated HCPs understood the importance of registers and how to use them, the pre-service training did not include recent changes made to vaccination guidelines (EPI prototype curriculum); hence, they lack the skills to track defaulters and monitor data.

Rowe et al. [31] also identified effective strategies for improving HCPs’ performance in LMICs. The authors gave a comprehensive evaluation of Health Care Provider Performance Review (HCPPR) strategies to improve healthcare providers’ performance in LMICs. The efficacy of HCP training, supervision, group problem-solving and community support were assessed. The study reported the importance and effectiveness of combining two or more strategies for improving HCPs’ practices in LMICs. HCP training, supervision and group problem-solving seem to be useful components in any strategy combination and implemented together proved more effective in improving HCPs’ competence and performance. The authors proposed monitoring of HCPs’ performance by implementing evidence-based strategies alongside local context knowledge for improvement and effective service delivery. This will enable the managers to know how well the strategies are working. Continuous monitoring and modification of implemented strategies were recommended in addition to the identification of gaps, and addressing these by modifying, removing or adding new strategies for improving HCPs’ performance.

## Discussion

Good-quality immunization data are crucial for effective policies on vaccine-preventable disease, and in particular the reduction and prevention of under-five morbidity and mortality. However, the task of collecting and managing these data in LMICs rests mostly on HCPs who often lack the necessary skills and competencies to effectively function in these roles [4]. Capacity building of HCPs on data management, completing immunization-monitoring chart, analysing and interpreting data and appropriate use of data for policy-making are key to ending vaccine-preventable diseases.

Findings from this scoping review provide evidence that shows both pre- and in-service trainings are essential for the development of HCPs’ competence in immunization data management, and analysis at all levels of the health system. The evidence shows that although pre-service training is fundamental, it does not adequately prepare HCPs, especially clinicians, with the necessary skills and competencies to collect, analyse and use data. This is due to a number of reasons, which include the inadequacies of the training curricula, which are not responsive to current needs, such as the changing trends in technology and preventive healthcare services, since the training curricula are often outdated [21–23, 30, 32].

More importantly, the training institutions are not adequately equipped to train HCPs on data skills as the majority of the tutors and clinical instructors at pre-service training institutes lack sufficient skills and knowledge to enable adequate translation of knowledge [10]. Continuous learning and development programmes that will increase educators’ knowledge to ensure optimal and current skills are often missing [21, 22, 24]. Although newly graduated nurses were capable of performing immunization work without in-service training, this review shows that they still require additional in-service training and mentoring/supportive supervision to function in their roles, particularly around documentation of patient care. Furthermore, lack of training equipment and the necessary tools and materials for clinical demonstration were absent, and in most cases, the time allocated for subject teaching is inadequate to ensure optimal knowledge translation.

Ward et al. [28] highlights various levels of responsibilities required by HCPs to effectively function in their roles, and through organizational assessment, data quality improvement (DQI) questionnaires and discussions with staff and data improvement teams (DITs), they identified external factors that affected vaccination data collection, management, analysis and use. These factors including poor motivation, new and untrained staff, staff absenteeism, lack of materials for recording and reporting data, competing priorities on staff time due to integration of services, inadequate supportive supervision for data quality, variable understanding and commitment by political or organizational leaders and competition with other public health initiatives for human and material resources are similar to results of other studies [4, 8, 33–35].

In the systematic review by Vasan et al. [30], which looked at different interventions to improve immunization data management in LMICs, in-service training, mentoring/supportive supervision was identified as important interventions in capacitating HCPs. For example, in Poy et al.’s [27] evaluation of the creation and implementation of a multidimensional monitoring tool (dashboard) for monitoring information on immunization-system performance in six African countries, an improvement in data quality between the first quarter of 2014 and fourth quarter of 2015 was reported, particularly for process indicators. In addition, Pappaioano et al. [29] reported an improvement in evidence-based public healthcare delivery following the prescribed data for decision-making (DDM) in-service training programme for mid-level health workers.

These findings are consistent with the study by Whittaker et al. [35], which looked at the competence of health information personal and described the core responsibilities of HIS personnel at different levels of the health system. They proposed that for the health system to generate good quality data, processes have to be put in place to ensure accurate data collection.

## Strengths and limitations

The strength of this review lies on the comprehensive nature of the search strategies used to identify the studies, which involves a range of methodologies that support exploratory review. The review extracted studies on HCPs’ capacity development on data collection and quality in immunization programmes in LMICs. However, the restrictions placed as a result of the selection criteria could potentially bias the findings. Our search terms were restricted to low- and middle-income countries and specifically on the expanded programme immunization (EPI), and the review relied only on open access articles or those accessible through the electronic database and search engines, and those articles published in English, thereby possibly omitting some useful articles on the subject.

## Conclusion

This scoping review has highlighted the challenges faced by HCPs in terms of competencies and skills to perform immunization data management tasks, which have been attributed to insufficient pre-service training. Even though pre-service training is fundamental in the skills acquisition of HCPs, they often require additional in-service training and supportive supervision to effectively function in immunization data management tasks.

Other issues around poor data management also stem from errors in processing data, limited use of already generated data, poor standardization of immunization data collection tools and processes such as registers, as well as ill-defined target population. These will require progressive implementation and uptake.

Immunization is one of the most cost-effective ways to save lives, improve health and ensure long-term prosperity. Achievement of the aim of the Astana Declaration on primary health care to meet all people’s health needs across the life course through comprehensive preventive and promotive care will require strong immunization programmes with well-trained and motivated workforce.

Continuous capacity development in immunization data management competencies such as data collection, analysis, interpretation, synthesis and data use, and efforts to improve data quality should be strengthened at all levels of the health system using the minimum health information competencies framework. Additionally, all new hires require orientation by employers in data management before they commence work.

Furthermore, there is a need for periodic review of the immunization-training curriculum in health training institutions, capacity development and retraining of tutors on the current trends in immunization data management such as the use of mobile technologies. The web district health information system (DHIS2) and tool like the WHO Immunization Information System (WIISE) are useful tools in improving immunization data management and use; however, these also require skilled workforce.

## Data Availability

Not applicable

## Abbreviations

AFRO: African Regional Office
CDC: Centers for Disease Control and Prevention
CHEW: Community health extension workers
CHO: Community health officers
CHW: Community health workers
CINAHL: Cumulative Index to Nursing and Allied Health Literature
DDM: Data for decision-making
DHIS2: District health information system 2
DITs: Data improvement teams
DQA: Data quality audit
DQI: Data quality improvement
EPI: Expanded Programme on Immunization
GAVI: Global Alliance for Vaccines and Immunization
HCPs: Healthcare providers
HCPPR: Health Care Provider Performance Review
HCWs: Healthcare workers
HIS: Health Information System
LMICs: Low and middle-income countries
MakSPH: Makerere University School of Public health
MeSH: Medical Subject Headings
MLM: Mid-level managers
NESI: Network for Education and Support in Immunization
PCC: Population–Concept–Context
SCHEW: Senior community health worker
SDG: Sustainable Development Goals
UHC: Universal health coverage
UNF: United Nations Foundation
WHO: World Health Organization
WIISE: WHO Immunization Information SystEm
